# Comparative outcomes of interventional and surgical management of abdominal lymphatic cysts in children

**DOI:** 10.3389/fped.2026.1825809

**Published:** 2026-06-03

**Authors:** Ying Jiang, Pan Jiao, Ke-Heng Deng, Jun Zhou, Li-li Huang, Zhaokun Guo

**Affiliations:** 1 Yichang Central People’s Hospital, Yichang, China; 2 Third People’s Hospital of Yichang City, Yichang, China

**Keywords:** abdominal lymphatic cyst, child, efficacy analysis, interventional therapy, surgical treatment

## Abstract

**Objective:**

To investigate the efficacy of interventional and surgical treatment for pediatric abdominal lymphatic cysts (ALC), and provide evidence for the selection of clinical treatment strategies.

**Methods:**

A retrospective analysis was conducted on the clinical data of children with ALC from January 2015 to December 2025. The patients were divided into an interventional group and a surgical group according to the initial treatment modality. Baseline data were collected for all patients meeting the inclusion criteria, encompassing variables such as gender, age, weight, pre-treatment cyst size, clinical manifestations, cyst location, cyst type, treatment method, treatment efficacy, postoperative complications, and recurrence rate. These data were subsequently compared across different groups.

**Results:**

The present study included a total of 21 pediatric patients diagnosed with abdominal lymphangioma, comprising 12 males and 9 females, with a mean age of 6.33 ± 2.54 years. Participants were allocated into two groups based on their initial treatment: the intervention group (*n* = 9) and the operation group (*n* = 12). The follow-up period post-discharge ranged from 6 months to 10 years, with a median duration of 4.2 years. No significant differences were observed between the two groups concerning gender, age, weight, cyst size, cyst location, or cyst type (*P* > 0.05). However, the incidence of acute abdomen was significantly higher in the operation group compared to the intervention group (6 cases *vs.* 0 cases). There were no statistically significant differences in the incidence of postoperative complications or recurrence rates (*P* > 0.05). Conversely, the operation group exhibited a significantly higher treatment efficacy rate (100% *vs.* 66.7%), hospitalization costs (14,418 ± 2,031 yuan *vs.* 11,075 ± 1,309 yuan), and length of hospital stay (8.5 ± 2.2 days *vs.* 5.3 ± 1.6 days) compared to the intervention group, with these differences being statistically significant (*P* < 0.05).

**Conclusions:**

Interventional therapy may serve as the primary treatment option for ALC, offering benefits such as minimal invasiveness, rapid recovery, and cost-effectiveness. Nevertheless, in the presence of complications like acute abdomen, surgical intervention is advised, with a preference for laparoscopic exploration.

## 1 Introduction

Abdominal lymphatic cyst (ALC) represents a rare benign vascular malformation in children, originating from aberrant embryonic development of the lymphatic system and predominantly presenting as intra-abdominal cystic space-occupying lesions (1, 2). The clinical manifestations are nonspecific, with abdominal pain and the presence of an abdominal mass being the primary symptoms. In certain cases, children may exhibit acute abdomen symptoms due to cyst compression, infection, rupture, or complicated intestinal torsion and obstruction (3, 4). Currently, the primary clinical interventions encompass interventional therapy and surgical treatment. Interventional therapy is gaining prominence in clinical practice due to its benefits of minimal invasiveness and expedited recovery; however, various cyst types have different responses to these treatment (5, 6). Each treatment modality presents distinct advantages and limitations. This study aims to evaluate and compare the efficacy, complication rates, and recurrence rates associated with these two therapeutic approaches, thereby offering a reference point for the development of personalized clinical treatment strategies.

## 2 Materials and methods

### 2.1 Study subjects and grouping

A retrospective analysis was performed on the clinical data of children with ALC admitted to the Department of Pediatric Surgery, Yichang Central People's Hospital from January 2015 to December 2025.

Inclusion criteria: (1) Confirmed ALC by imaging or pathological examination; (2) Received interventional or surgical treatment in our hospital; (3) Complete clinical data and follow-up duration ≥6 months.

Exclusion criteria: (1) Complicated with other congenital malformations or severe organ dysfunction; (2) Lost to follow-up or incomplete clinical data; (3) History of previous abdominal surgery.

This study received approval from the Ethics Committee of Yichang Central People's Hospital. A standardized criterion for treatment decision-making regarding ALC was formulated in our hospital, with treatment plans being determined through collaborative discussions between pediatric surgeons and interventional radiologists. Over the extended duration of the study, the criteria for treatment decision-making evolved. In the last 5 years, the indications for interventional therapy have included the presence of cysts with appropriate puncture access, the absence of contraindications to interventional therapy, and the requirement that parents fully understand the associated treatment risks and provide informed consent for interventional therapy.

**Figure 1:**
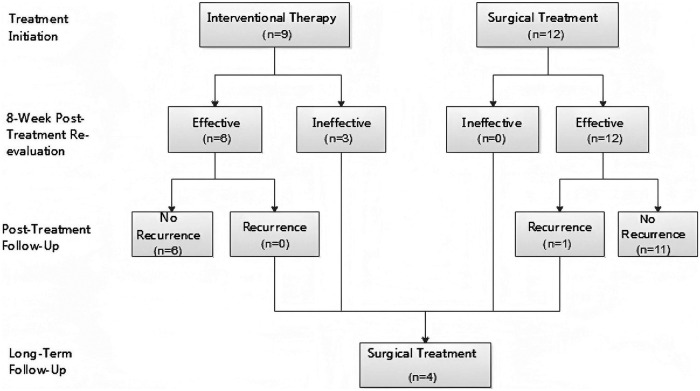
Treatment flowchart for pediatric abdominal lymphatic cycts.

Criteria for surgical intervention include unsuccessful or recurrent interventional therapy, ineligibility for interventional procedures (such as cases complicated by acute abdomen or lack of suitable puncture access), and parental preference for surgical treatment over interventional methods. Additionally, a standardized treatment flowchart for pediatric ALC was developed, ensuring that all pediatric patients received care in accordance with this protocol (Figure 1).

### 2.2 Clinical manifestations and imaging examinations

All children underwent preoperative ultrasound examination. CT and/or MRI examinations were performed in a subset of patients to clarify the location, size, cyst type of the lesions, and their anatomical relationships with surrounding blood vessels and organs. Measurement of cyst volume before and after surgery was performed separately by an ultrasound physician and a radiologist, and re-measured by a pediatric surgeon based on CT or MRI images. The median value of the three sets of data was taken as the final result.

### 2.3 Treatment methods

#### 2.3.1 Interventional therapy

**Figure 2:**
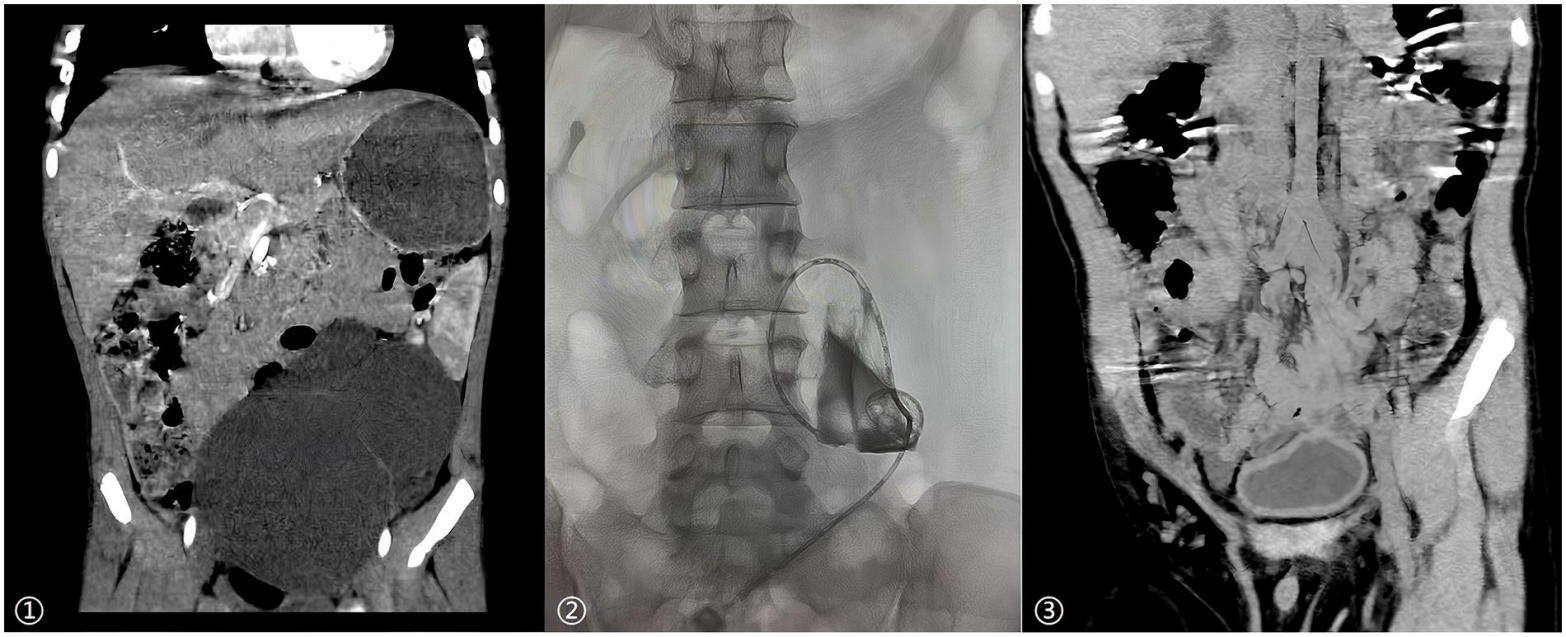
(1) preoperative coronal CT image showing a large cystic lesion in the pelvis. (2) Intraprocedural image obtained during interventional drainage: after percutaneous puncture, a drainage catheter was placed and contrast agent was injected, opacifying the cyst cavity. (3) Postoperative 8-week follow-up coronal CT image demonstrates significant interval reduction of the cystic lesion.

Macrocystic and mixed type children received interventional therapy; microcystic type was not treated with interventional therapy due to tiny cyst cavities and difficulty in accurate puncture. All operations were performed under general anesthesia and DSA guidance. Doxycycline injection was used as the sclerosant, mixed with normal saline and water-soluble contrast agent to prepare a 10 mg/mL solution. The maximum diameter and optimal puncture path of the cyst were identified under DSA, while avoiding important structures such as blood vessels and organs. The puncture needle was slowly advanced along the path, with aspiration during advancement. After confirming entry into the cyst cavity, the needle core was withdrawn, a drainage tube was inserted, connected to a three-way stopcock and syringe, and the cyst fluid was completely aspirated. The volume and properties (clear, turbid or chylous) of the aspirated fluid were recorded. For multilocular cysts, a drainage tube was only placed in the largest cyst, and remaining large cysts were punctured and aspirated separately with needles. DSA angiography was performed to confirm the drainage tube was still in the cyst cavity. Then, according to the aspirated volume, the prepared doxycycline sclerosant was injected at a ratio of 1/3–1/2, with a single dose not exceeding 20 mg/kg. DSA was continuously monitored during injection to avoid drug extravasation; if resistance was high, the injection volume was appropriately reduced to prevent cyst rupture. On the first postoperative day, sclerosant was injected again through the drainage tube in the ward; if the preoperative cyst diameter was >5 cm, a second injection was given on the second postoperative day. After each sclerosant injection, the drainage tube was clamped for 6 h and then opened for drainage. The drainage tube was removed after the last treatment. Follow-up imaging using ultrasound, CT, or MRI was performed 8 weeks postoperatively. If the cyst volume decreased by < 25% or clinical symptoms did not disappear, the treatment was considered ineffective and converted to surgical treatment (Figure 2).

#### 2.3.2 Doxycycline safety monitoring protocol

(1) Local complication monitoring: Daily monitoring of redness, swelling, exudation and pain at the puncture site from postoperative period util extubation; recording of abdominal drainage volume and color; monitoring for signs of peritonitis; recording of severity and duration of abdominal pain. (2) Systemic complication monitoring: Body temperature was monitored every 4 h within 72 h after surgery to screen for fever; routine blood tests were performed and inflammatory markers were measured to exclude infection. (3) Special monitoring for tooth pigmentation: Children ≤ 8 years old underwent annual oral examinations in the department of stomatology after surgery to observe enamel and color changes until the end of follow-up. (4) Other systemic reactions: Monitoring of gastrointestinal reactions such as nausea, vomiting and diarrhea.

#### 2.3.3 Surgical approach

Laparoscopic exploration was prioritized for children presenting with acute abdomen and highly suspected intestinal torsion/obstruction; converted to open surgery if the laparoscopic procedure was difficult due to large cyst volume, the presence of large surrounding blood vessels or severe adhesions.

#### 2.3.4 Surgical procedures

**Figure 3:**
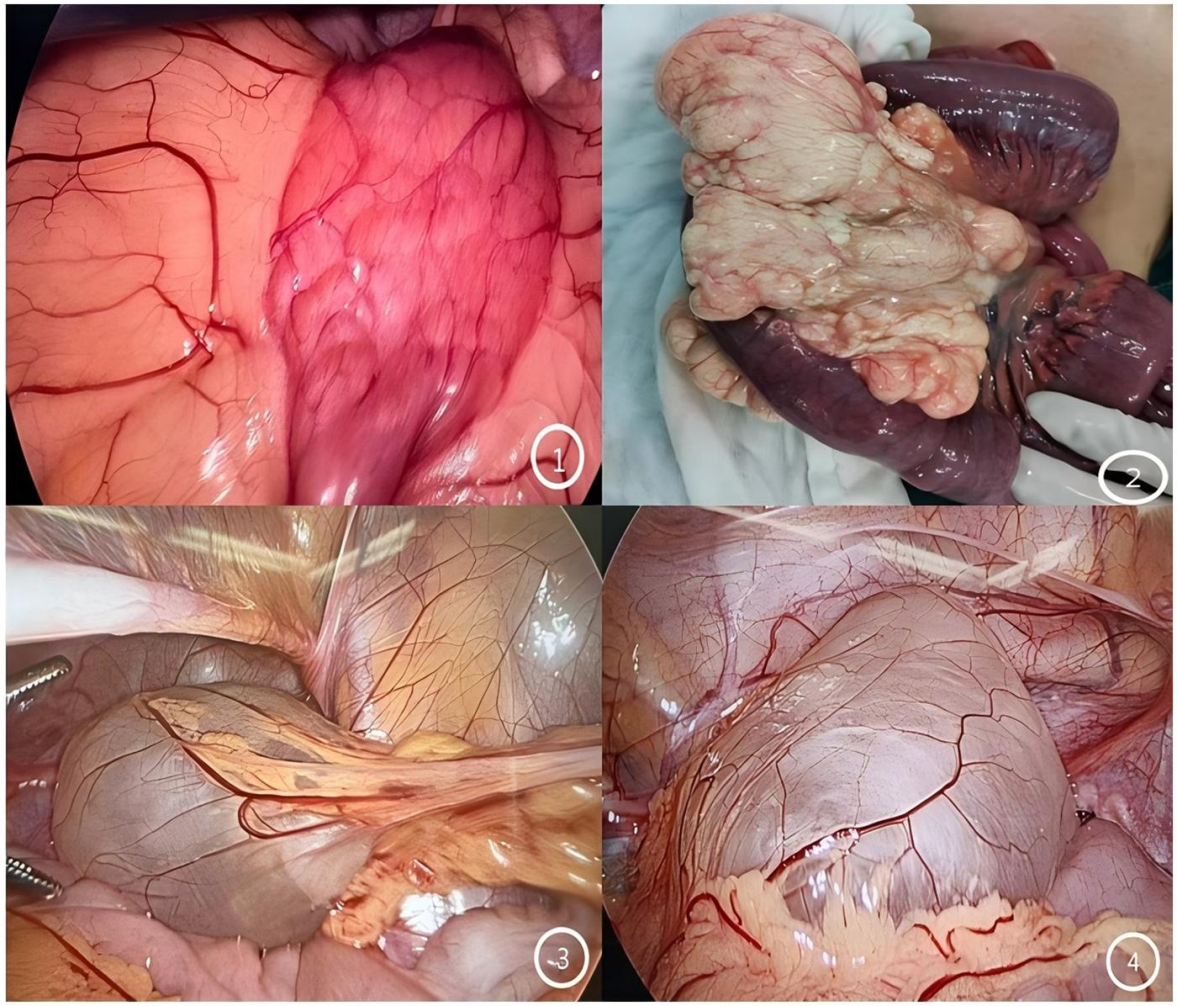
Small mesenteric dumbbell cyst, cyst combined with intestinal resection; 2: small mesenteric dumbbell cyst and small intestine torsion, cyst combined with intestinal resection; 3: greater omentum cyst, cyst complete resection; 4: other abdominal space cysts: complete resection of the cyst.

(1) Complete cyst resection: Omental cyst, pedicled mesenteric cyst of the small intestine, and other intra-abdominal space cysts. (2) Cyst resection combined with intestinal resection: dumbbell-shaped mesenteric cyst of the small intestine, mesenteric cyst of the small intestine complicated by intestinal torsion; cyst puncture and decompression, extension of the umbilical incision, exteriorization for resection and intestinal anastomosis. (3) Partial cyst resection: Indicated when the cyst base involves mesenteric vessels and cannot be completely resected; most cyst tissues were removed, residual cavity inner wall was treated by electrocautery, and a drainage tube was placed (Figure 3).

### 2.4 Follow-up and efficacy evaluation

The follow-up process involved outpatient reexaminations, as well as ultrasound, CT, or MRI assessments, complemented by telephone interviews. The overall duration of follow-up ranged from 6 months to 10 years, with a median duration of 4.2 years. Specifically, follow-up for adverse events related to doxycycline was conducted for 2 years post-surgery, while monitoring for tooth pigmentation in young children continued until the complete eruption of permanent teeth. The follow-up schedule entailed outpatient reexaminations for all children at 8 weeks post-surgery, followed by reexaminations every 3 months during the first year and every 6 months thereafter. Additionally, telephone follow-ups were conducted monthly to document any complications or adverse events.

Criteria for Evaluating Treatment Efficacy: (1) Effective: A reduction in cyst volume by 90% or more, accompanied by an improvement in clinical symptoms following treatment. (2) Ineffective: A reduction in cyst volume by less than 25% or the persistence of clinical symptoms post-treatment. (3) Recurrent: Initial disappearance or reduction of the cyst post-treatment, followed by reappearance or recurrence of symptoms during the follow-up period. Data were collected on hospitalization costs, duration of hospital stay, efficacy rate, recurrence rate, complications associated with the two primary treatments, and the therapeutic outcomes in crossover subgroups.

### 2.5 Statistical analysis

Data analysis was conducted using SPSS version 21.0. Measurement data were expressed as mean ± standard deviation, and comparison between groups was performed by t-test. Categorical variables were presented as frequencies (percentages), with comparisons between groups performed using *χ*^2^ test or Fisher's exact test. A *p*-value of less than 0.05 was considered indicative of statistical significance.

## 3 Results

### 3.1 Baseline data description

A cohort of 21 pediatric patients diagnosed with ALC was enrolled in the study, comprising 12 males and 9 females, with a mean age of 6.33 ± 2.54 years (range: 1–14 years) and an average body weight of 21.07 ± 5.38 kg. Based on the initial treatment approach, participants were categorized into an interventional group (*n* = 9) and a surgical group (*n* = 12) (Table 1). Clinical presentations included abdominal pain in 6 cases (28.6%), abdominal mass in 14 cases (66.7%), and anemia in 1 case (4.7%). All subjects underwent preoperative ultrasound assessment; additionally, 17 patients received CT scans and 9 underwent MRI examinations (with 5 patients receiving both CT and MRI) to determine the precise location, size, cyst type, and anatomical relationships with adjacent blood vessels and organs. The lesions were categorized according to cyst diameter and morphology as follows: macrocystic (maximum cyst diameter ≥1 cm) in 15 cases (71.4%), microcystic (maximum cyst diameter <1 cm) in 2 cases (9.5%), and mixed type (predominantly microcystic with dominant cysts suitable for puncture treatment) in 4 cases (19.1%). The baseline characteristics and distribution of the disease spectrum between the two groups are presented in Tables 1, 2.

**Table 1:** A comparative analysis of baseline data in pediatric patients with abdominal lymphatic cysts undergoing interventional therapy versus surgical treatment.

Items	Interventional Group (*n* = 9)	Surgical Group (*n* = 12)	F/*χ*^2^ Value	*P*-Value
Gender (Male/Female, cases)	5/4	7/5	0.010	0.920
Age (mean ± sd, years)	6.25 ± 2.47	6.41 ± 2.58	0.113	0.921
Body Weight (mean ± sd, kg)	21.39 ± 5.39	20.92 ± 5.41	0.221	0.385
Cyst Size before Treatment (mean ± sd, cm)	347.21 ± 109.38	317.54 ± 142.77	0.386	0.817
Complicated with Acute Abdomen (Yes/No, cases)	0/9	6/6	Fisher	0.015
Complicated with Intracystic Hemorrhage (Yes/No, cases)	0/9	1/11	Fisher	0.470

The difference between the interventional group and the surgical group in the presence of acute abdomen was statistically significant.

**Table 2:** An analysis of the Spectrum of diseases in pediatric patients with abdominal lymphatic cysts undergoing interventional therapy compared to surgical treatment.

Items	Interventional group (*n* = 9)	Surgical group (*n* = 12)	*P*-Value
Cyst Location (cases)			
Small Intestinal Mesentery	4	9	0.946
Greater Omentum	3	2	0.609
Other Abdominal Spaces	2	1	0.384
Cyst Type (cases)			
MAC	6	9	0.785
MIC	0	2	0.470
CBC	3	1	0.619

MAC, macrocystic type; MIC, microcystic type; CBC, combined type.

### 3.2 Comparison between groups

No statistically significant differences were observed between the two groups concerning gender, age, body weight, pre-treatment cyst size, presence of complicated intracystic hemorrhage, cyst location, or cyst type (*P* > 0.05) (Tables 1, 2). Similarly, no significant differences were found in the rates of complications or recurrence between the groups (*P* > 0.05). However, the surgical group exhibited significantly higher rates of complicated acute abdomen, hospitalization costs, length of hospital stay, and effectiveness rate compared to the interventional group, with these differences reaching statistical significance (*P* < 0.05) (Table 1 and Table 3).

**Table 3:** A comparative study of the therapeutic outcomes in pediatric patients with abdominal lymphatic cysts treated via interventional therapy versus surgical intervention.

Items	Interventional group (*n* = 9)	Surgical group (*n* = 12)	F/χ^2^ Value	*P*-Value
Hospitalization Cost (x ± s, RMB)	11,075 ± 1,309	14,418 ± 2,031	4.621	<0.001
Hospital Stay (x ± s, days)	5.3 ± 1.6	8.5 ± 2.2	3.957	0.001
Effective Rate			4.743	0.030
Macrocystic Type (MAC)	6/6	9/9	-	-
Microcystic Type (MIC)	-	2/2	-	-
Combined Type (CBC)	0/3	1/1	-	-
Total (%)	66.7	100	-	-
Recurrence Rate			Fisher	1.000
Macrocystic Type (MAC)	0/6	0/9	-	-
Microcystic Type (MIC)	-	1/2	-	-
Combined Type (CBC)	-	0/1	-	-
Total (%)	0	8.33	-	-
Complication Rate			Fisher	0.609
Macrocystic Type (MAC)	2/6	1/9	-	-
Microcystic Type (MIC)	-	0/2	-	-
Combined Type (CBC)	0/3	1/1	-	-
Total (%)	22.2	16.7	-	-

MAC, macrocystic type; MIC, microcystic type; CBC, combined type. Statistically significant differences were observed between the interventional and surgical groups in terms of hospitalization costs, duration of hospital stay, and treatment efficacy rates.

### 3.3 Follow-up details of doxycycline adverse reactions

The follow-up period following discharge varied from 6 months to 10 years, with a median duration of 4.2 years. Of the 9 children in the interventional group who received doxycycline as a sclerosant, 2 cases involving macrocystic conditions experienced sclerosant leakage into the abdominal cavity post-surgery. This leakage manifested as abdominal pain and fever, which subsided with bed rest, anti-infective measures, and symptomatic treatment (Clavien-Dindo grade II). No severe complications, such as systemic infection, were observed. Furthermore, none of the children aged 8 years or younger exhibited characteristic adverse reactions to tetracyclines, such as tooth pigmentation, after a 2-year follow-up period.

## 4 Discussion

Abdominal lymphatic cysts represent an uncommon benign lesion in pediatric populations, with their pathogenesis attributed to aberrant embryonic development of the lymphatic system. These cysts can manifest in any region abundant in lymphoid tissue within the abdominal cavity, with a predilection for the small intestinal mesentery and greater omentum (7). Due to their indolent growth, most cysts remain asymptomatic during the initial stages. However, as they enlarge or if complications arise, they may present with symptoms such as abdominal pain and palpable abdominal mass. In certain cases, children may require urgent medical attention due to acute abdominal conditions (8, 9). In this study, the incidence of complicated acute abdomen was significantly greater in the surgical cohort compared to the interventional cohort. This finding indicates that acute abdomen is a critical indication for the initial surgical management of pediatric ALC, aligning with the standardized treatment decision-making criteria established at our institution.

Acute abdomen in pediatric abdominal lymphatic cysts arises from cyst torsion, rupture, infection, or mechanical complications like intussusception. Torsion causes ischemia and severe pain; rupture leads to chemical peritonitis; infection results in abscess formation; and large cysts may trigger bowel obstruction. Clinically, children present with sudden severe abdominal pain, guarding, vomiting, and fever, potentially progressing to shock. Imaging (ultrasound, CT) reveals cyst wall thickening, septations, fluid, or obstruction signs. Recognizing these triggers is essential for timely intervention (10–12). Consequently, in light of this situation, the immediate implementation of surgical treatment is crucial (13, 14). This approach aligns closely with the diagnostic and therapeutic guidelines proposed in this study. Recurrence rates vary depending on the completeness of cyst excision; complete resection is associated with a 5%–10% recurrence rate, whereas partial excision can result in recurrence rates as high as 20%–30% (15, 16). This underscores the importance of achieving total cyst removal when feasible.

Our data show that surgical intervention has a highly favorable prognosis, with a 100% effective rate, surpassing the interventional group, and a low recurrence rate of 8.33%. Surgery effectively removes lesions, improves outcomes, and reduces recurrence risk. It also addresses complications like cysts adhering to intestinal tubes, invading blood vessels, or causing intestinal torsion/obstruction. In this study, six children with acute abdomen recovered well post-surgery without major complications (17). Laparoscopic exploration is beneficial for suspected intestinal torsion/obstruction in children due to its minimally invasive nature and clear visibility, allowing for prompt diagnosis and treatment. Among 12 surgical cases, all initially underwent laparoscopy, but three required conversions to open surgery due to large cysts and extensive intestinal involvement.

In addition to cases involving abdominal mass and acute abdomen, we treated one child presenting with anemia, who was diagnosed with ALC complicated by intracystic hemorrhage, as confirmed through abdominal ultrasound, CT and MRI, and finally cured by surgical treatment. The diagnosis of ALC depends on a combination of ultrasound, CT and MRI. All children underwent abdominal ultrasound in the outpatient setting; if ALC was suspected, further diagnostic evaluation with abdominal CT or MRI was conducted post-admission. CT and MRI can provide three-dimensional imaging that aids in delineating the anatomical relationship between blood vessels and cysts, and they are also important for reevaluating cyst volume changes after treatment.

As a minimally invasive treatment, interventional therapy has the advantages of minimal invasiveness, rapid recovery, low cost and no scar. Its core operations are accurate puncture, complete drainage and effective sclerosis (18, 19). In this study, it achieved a 100% success rate for macrocystic ALC with no recurrence, aligning with past research (20). The treatment works by using a sclerosant to damage lymphatic endothelial cells, causing fibrosis and closure of the cyst. Meanwhile, DSA-guided puncture combined with water-soluble contrast agent to monitor drug distribution effectively improved operational safety and avoided injury to blood vessels and organs. However, the success of interventional therapy depends on the cyst type. In cases involving mixed-type and microcapsule-type formations, the incomplete penetration of the sclerosing agent results in the inability to puncture the microcapsules, necessitating surgical intervention (21). Interventional therapy should adhere to strict guidelines and is not recommended as the first option for children lacking suitable puncture access or with acute abdominal complications.

The study confirmed doxycycline's safety as a local sclerosing agent for intra-abdominal lymphatic cysts in children, with no systemic complications or dental staining observed. Only two cases of sclerosant leakage into the peritoneal cavity occurred, resolving with short-term conservative treatment. Doxycycline's local application targets the cyst lining, minimizing systemic absorption and avoiding typical tetracycline side effects, aligning with existing research (2, 20, 22, 23). We also observed that researchers have used new interventional techniques like bleomycin electrosclerotherapy and percutaneous thermal ablation for vascular malformations unresponsive to traditional treatments (24). This highlights the need to broaden treatment options. Sirolimus has shown effectiveness in managing complex lymphatic malformations by inhibiting the mTOR/PI3 K pathway and reducing VEGF activity, proving beneficial for cases resistant to sclerotherapy and those involving critical areas (25).

Both treatment methods led to complications. In the interventional group, two cases experienced macrocystic lesions with sclerosant leakage into the abdominal cavity, causing pain and fever, which improved with bed rest, anti-infection therapy, and supportive treatment (Clavien-Dindo grade II). In the surgical group, one case involved an intraoperative injury to the small intestinal wall, repaired during surgery (Clavien-Dindo grade III), and another case had postoperative chylous leakage, which resolved after two weeks of conservative treatment (Clavien-Dindo grade II). The complication rate was 22.2% for the interventional group and 16.7% for the surgical group, with no significant difference between them. All complications resolved with symptomatic treatment, showing both treatments are safe. However, surgery involves more trauma, longer hospital stays, and higher costs. In cases with dense adhesions, intestinal resection may be needed, affecting function. Thus, surgical indications should be strictly controlled, and interventional therapy remains the preferred option for macrocystic children without acute abdomen who qualify for it.

This study has limitations: it's a single-center retrospective study with a small sample size, potentially leading to selection bias due to early-stage interventional experience. The follow-up periods vary, with some being short, possibly affecting long-term efficacy evaluation. Additionally, no stratified analysis of sclerosant dose or injection frequency was conducted. Future research should include multicenter, large-sample prospective studies to improve treatment plans.

## 5 Conclusions

Pediatric ALC treatment should be tailored based on cyst type and symptoms. For macrocystic ALC without acute abdomen, interventional therapy is preferred due to its precision, minimal invasiveness, quick recovery, and low cost. However, for microcystic and mixed ALC, surgery is advised. Surgery is suitable for all ALC types, especially if acute abdomen or suspected intestinal torsion/obstruction is present, with laparoscopic exploration as a priority.

## Data Availability

The raw data supporting the conclusions of this article will be made available by the authors, without undue reservation.
